# Construction of a ceRNA-based lncRNA-mRNA network to identify functional lncRNAs in polycystic ovarian syndrome

**DOI:** 10.18632/aging.202659

**Published:** 2021-03-10

**Authors:** Yue Ma, Linna Ma, Yurong Cao, Jun Zhai

**Affiliations:** 1 Center for Reproductive Medicine, The First Affiliated Hospital of Zhengzhou University, Zhengzhou, China; 2 Henan Key Laboratory of Reproduction and Genetics, The First Affiliated Hospital of Zhengzhou University, Zhengzhou, China; 3 Henan Provincial Obstetrical and Gynecological Diseases (Reproductive Medicine) Clinical Research Center, The First Affiliated Hospital of Zhengzhou University, Zhengzhou, China

**Keywords:** polycystic ovary syndrome, bioinformatics, lncRNA, ceRNA, biomarkers

## Abstract

Polycystic ovary syndrome (PCOS) is a prevalent endocrine and metabolic disorder in women of childbearing age. Recent studies suggest important roles for lncRNAs in PCOS development. Based on the hypothesis that lncRNAs are able to regulate mRNA functions by competitive binding to shared miRNAs, the present work sought to construct a PCOS-related lncRNA-mRNA network (PCLMN) to identify key lncRNAs with dysregulated expression and potential prognostic and therapeutic relevance. A global background network was constructed after retrieving lncRNA-miRNA and miRNA-mRNA pairs from the lncRNASNP2, miRTarBase and StarBase databases. Based on gene expression profiles from ovarian granulosa cells from PCOS patients and controls in the GEO’s GSE95728 dataset, the PCLMN was then constructed by applying hypergeometric testing. Using topological analysis, we identified 3 lncRNAs (LINC00667, AC073172.1 and H19) ranking within the top-ten gene lists for all three centrality measures. We then explored their subcellular localization, performed functional module analyses, and identified 4 sex hormone-related transcription factors as potential regulators of their expression. Significant associations with inflammation, oxidative stress, and apoptosis-related processes and pathways were revealed for the key lncRNAs in our PCMLN. Further studies verifying the mRNA/lncRNA relationships identified herein are needed to clarify their clinical significance.

## INTRODUCTION

Polycystic ovary syndrome (PCOS) is one of the most prevalent endocrine and metabolic disorders, affecting up to one in five women of childbearing age [1]. Its cardinal features are hyperandrogenism, menstrual irregularity, and polycystic ovary morphology [2]. PCOS is associated with insulin resistance (IR), metabolic syndrome, increased risk of endometrial cancer, ovulatory dysfunction, infertility, pregnancy complications, type 2 diabetes, and cardiovascular disease [3–7]. Although the pathogenesis of PCOS is complex and remains unclear, there is growing scientific consensus in that genetic factors play a key role in PCOS occurrence and development.

Long non-coding RNAs (lncRNAs) represent a class of non-coding RNA transcripts greater than 200 nucleotides in length [8]. Although lncRNAs lack protein-encoding capacity, an increasing body of evidence suggests that they act a pivotal part in many biological processes, including genetic imprinting, X-chromosome inactivation, transcriptional and post-transcriptional regulation, recruitment of epigenetic modifiers, control of mRNA decay, organelle biogenesis, and subcellular trafficking, among others [9, 10]. Accumulating evidence also suggests that lncRNA dysregulation is closely associated with numerous human diseases, including PCOS and PCOS-related conditions. For instance, results from microarray analyses showed that some lncRNAs were abnormally expressed in the granulosa cells of PCOS patients, which suggested their involvement in PCOS development [10]. Examples include HCG26, an lncRNA that participates in the regulation of ovarian granulosa cell proliferation and steroidogenesis [11], and RP11-151A6.4, which was found to be upregulated in granulosa cells from PCOS patients and suggested to promote insulin resistance, androgen excess, and adipose dysfunction [12]. However, only a few lncRNAs have been so far verifiably related to PCOS.

The competitive endogenous RNA (ceRNA) hypothesis describes a regulatory network involving mutually interacting protein-coding (mRNA) and non-coding (e.g. lncRNA, miRNA, etc.) transcripts to modulate gene and protein expression, A central tenet of the ceRNA hypothesis is that lncRNAs can regulate other RNA transcripts by competitive binding to miRNAs via shared miRNA response elements [13]. ceRNA activities have been confirmed to influence the development of several diseases, and ceRNA networks have been built for lung cancer [14], cardiac hypertrophy [15], and implantation failure [16], among other conditions. Considering the potential relevance of lncRNAs in the regulation of PCOS through the ceRNA mechanism, in this study we used bioinformatics tools to construct a PCOS-related lncRNA-mRNA network (PCLMN) to identify key lncRNAs that might impact the development and serve as biomarkers of PCOS.

## RESULTS

### Identification of differentially expressed lncRNAs and mRNAs

LncRNA and mRNA expression profiles from granulosa cells from seven PCOS patients and seven controls were retrieved from the GSE95728 dataset available on the NCBI-GEO repository. Based on FC > 2 and adjusted P < 0.05, 86 differentially expressed lncRNAs (DELs) and 112 differentially expressed mRNAs (DEMs) were identified.

### Construction of a global lncRNA-miRNA-mRNA triple network

A total of 50,2653 miRNA-mRNA interaction pairs were downloaded from miRTarBase and starBase. Subsequently, the lncRNASNP2 tool was applied to select candidate miRNAs targeting the 86 DELs identified in the GSE95728 dataset. DELs’ sequences were obtained from UCSC Genome Browser, and miRNA/lncRNA associations with a prediction score > 160 and binding energy < -20 were selected. A total of 66,059 miRNA-lncRNA interaction pairs were thus obtained. All selected miRNA-mRNA and miRNA-lncRNA pairs were then merged to construct a global triple network which was used as a background network to construct the PCLMN.

### Construction of the PCOS-related lncRNA-mRNA network

**Figure 1:**
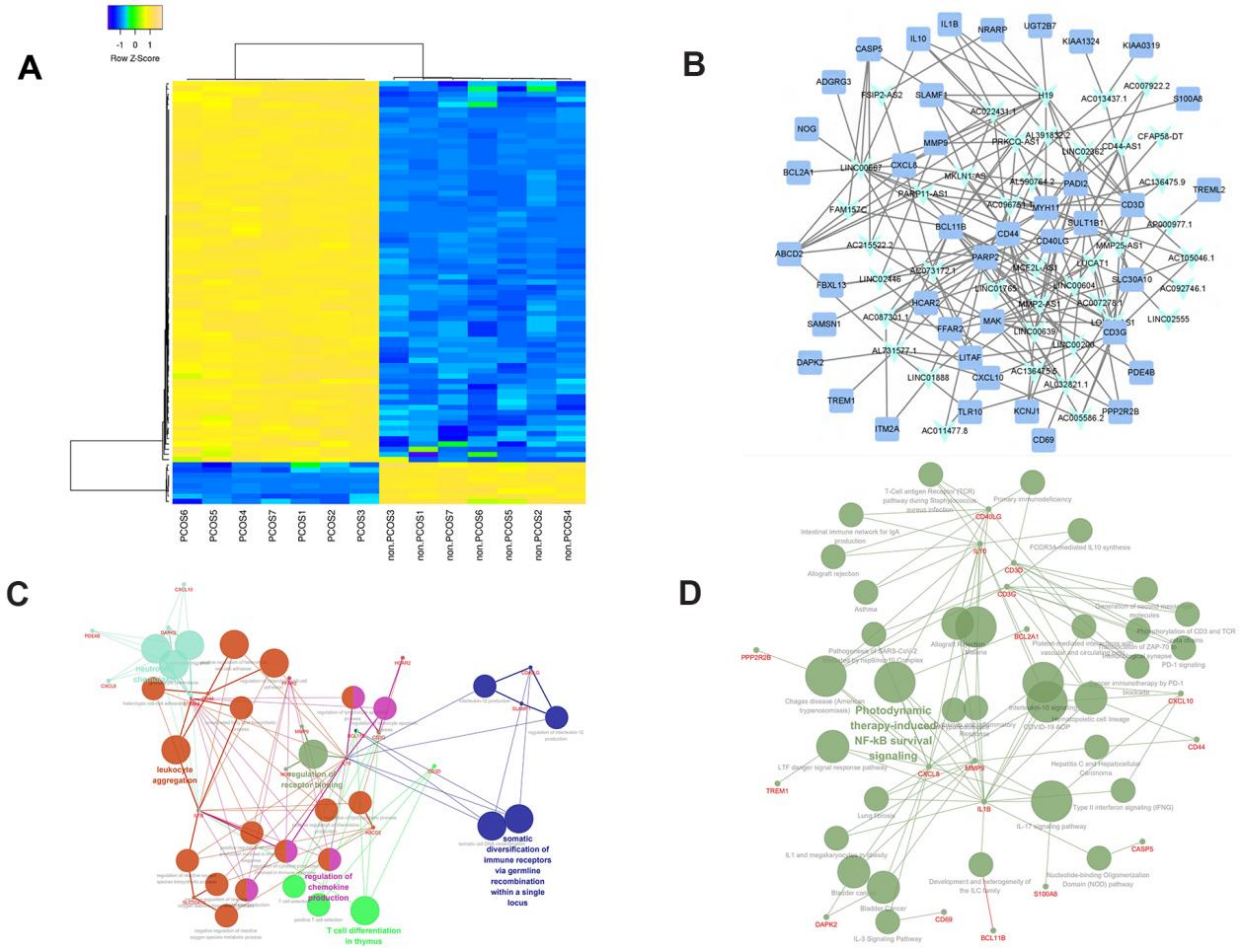
**Clustering and enrichment analysis of the PCOS-related lncRNA-mRNA network (PCLMN).** (**A**) Unsupervised clustering of PCLMN genes. (**B**) PCLMN visualization. Light-blue arrowheads indicate lncRNAs and blue squares indicate mRNAs. Grey edges indicate lncRNA-mRNA interactions. (**C**) Network map of enriched GO terms in the PCLMN. (**D**) Network map of enriched KEGG pathways in the PCLMN.

After mapping all the DELs and DEMs into the global triple network, a hypergeometric test was performed to extract 334 lncRNA-miRNA-mRNA triplets. From these, 41 lncRNAs and 41mRNAs, linked by 203 edges, were chosen to construct the PCLMN (Figure 1B).

### Hierarchical clustering and functional characteristics of the PCLMN

**Figure 2:**
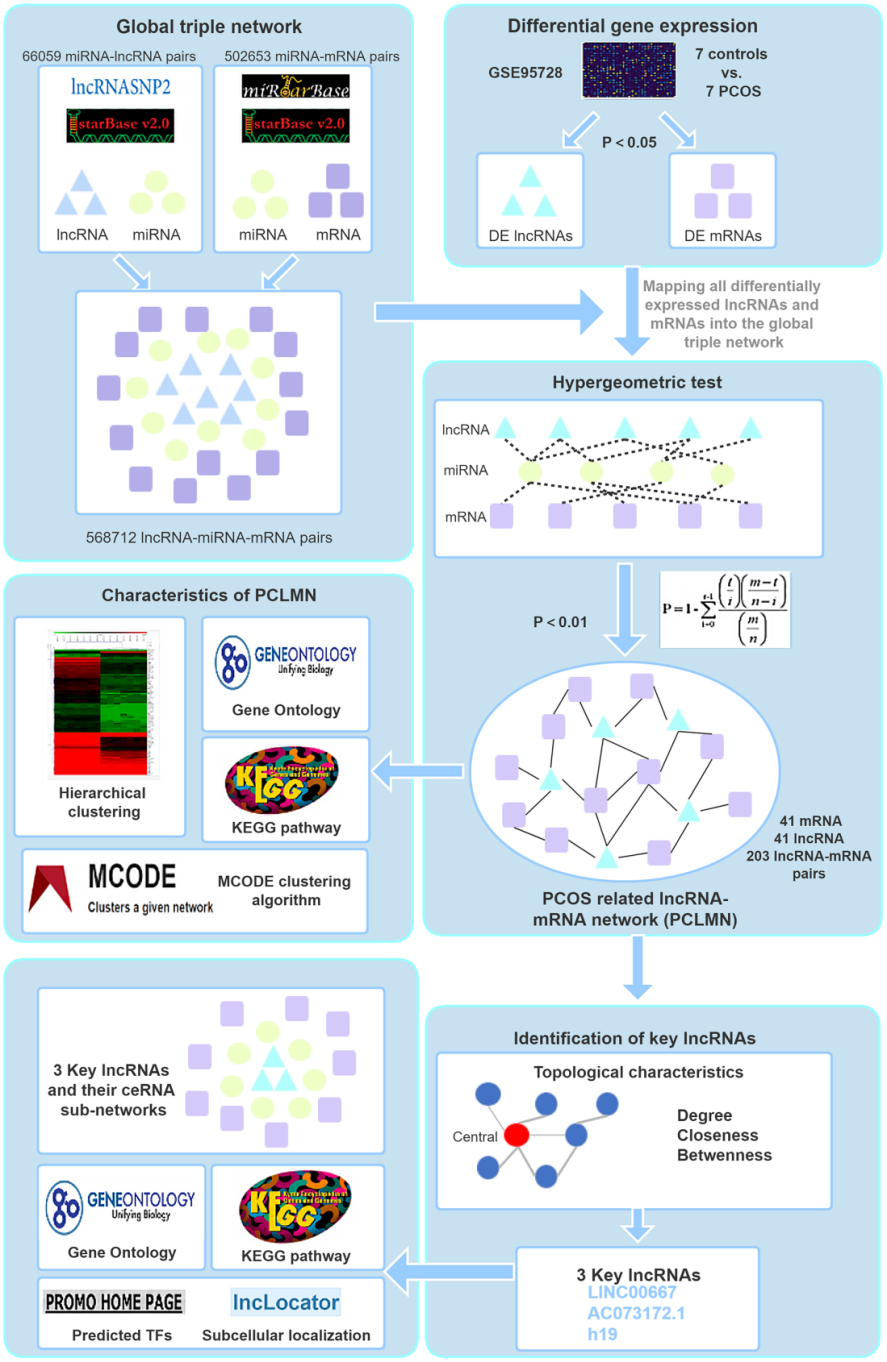
**Study workflow.** First, we constructed a global background network based on predicted lncRNA-miRNA and miRNA-mRNA pairs. Second, we applied a hypergeometric test to construct the PCLMN and performed network topology analysis to determine the lncRNAs with the highest centroid variability. Lastly, we explored the subcellular localization of the key lncRNAs thus identified, performed functional module analyses, and identified putative transcription factors regulating the expression of the candidate lncRNAs.

The 82 differentially expressed genes (DEGs) included in the PCLMN were grouped by unsupervised hierarchical clustering, suggesting distinct gene expression profiles for control and PCOS (Figure 1A). We next performed GO classification and KEGG pathway analysis on the DELs and DEMs in the PCLMN. GO analysis results indicated significant enrichment in 27 GO terms (P≤0.05, Benjamini-Hochberg-corrected) as listed in Figure 1C and Table 1. Meanwhile, as shown in Figure 2 and Table 2, 13 pathways were found to be enriched (P≤0.05) in the target genes.

**Table 1:** Enriched GO terms in the PCLMN.

**GO term**	**P-value**	**Associated genes (%)**
regulation of receptor binding	2.67E-05	10.71
T cell selection	1.64E-04	5.88
positive T cell selection	6.78E-05	7.89
T cell differentiation in thymus	2.20E-05	5.06
granulocyte chemotaxis	4.15E-07	4.32
neutrophil migration	2.80E-07	4.62
neutrophil chemotaxis	1.09E-07	5.41
interleukin-12 production	3.23E-04	4.69
regulation of interleukin-12 production	2.94E-04	4.84
somatic diversification of immune receptors	4.75E-04	4.11
somatic cell DNA recombination	4.75E-04	4.11
chemokine production	4.93E-05	4.12
regulation of cytokine production involved in immune response	5.56E-05	4.00
regulation of chemokine production	4.01E-05	4.35
regulation of leukocyte apoptotic process	4.01E-05	4.35
regulation of lymphocyte apoptotic process	2.67E-04	5.00
heterotypic cell-cell adhesion	3.38E-04	4.62
chemokine production	4.93E-05	4.12
regulation of lipid catabolic process	3.38E-04	4.62
leukocyte aggregation	3.03E-06	21.43
regulation of reactive oxygen species biosynthetic process	5.56E-05	4.00
negative regulation of reactive oxygen species metabolic process	3.53E-04	4.55
regulation of cytokine production involved in immune response	5.56E-05	4.00
regulation of chemokine production	4.01E-05	4.35
regulation of heterotypic cell-cell adhesion	2.39E-05	11.11
negative regulation of reactive oxygen species biosynthetic process	4.42E-05	9.09
positive regulation of cytokine production involved in immune response	3.69E-04	4.48
positive regulation of chemokine production	4.03E-04	4.35
positive regulation of heterotypic cell-cell adhesion	4.65E-06	18.75
unsaturated fatty acid biosynthetic process	3.38E-04	4.62
regulation of lymphocyte apoptotic process	2.67E-04	5.00

**Table 2:** Enriched KEGG pathways in the PCLMN.

**GO term**	**P-value**	**Associated genes%**
Hematopoietic cell lineage	2.09E-04	4.04
IL-17 signaling pathway	8.26E-06	5.32
Intestinal immune network for IgA production	9.35E-03	4.08
Chagas disease (American trypanosomiasis)	5.22E-07	5.88
African trypanosomiasis	5.42E-03	5.41
Malaria	1.42E-05	8.00
Bladder cancer	2.43E-04	7.32
Asthma	3.82E-03	6.45
Allograft rejection	5.71E-03	5.26
Primary immunodeficiency	5.71E-03	5.26
Phosphorylation of CD3 and TCR zeta chains	1.93E-03	9.09
Translocation of ZAP-70 to Immunological synapse	1.44E-03	10.53
Generation of second messenger molecules	4.59E-03	5.88
PD-1 signaling	2.11E-03	8.70
Interleukin-10 signaling	1.11E-05	8.51
FCGR3A-mediated IL10 synthesis	7.26E-03	4.65
Nucleotide-binding Oligomerization Domain (NOD) pathway	6.62E-03	4.88
Allograft Rejection	1.44E-04	4.44
Bladder Cancer	2.43E-04	7.32
IL-3 Signaling Pathway	9.35E-03	4.08
IL1 and megakaryocytes in obesity	2.50E-03	8.00
Photodynamic therapy-induced NF-kB survival signaling	5.56E-08	14.29
Lung fibrosis	8.64E-04	4.76
Hepatitis C and Hepatocellular Carcinoma	9.72E-03	4.00
T-Cell antigen Receptor pathway during Staphylococcus aureus infection	8.25E-04	4.84
Development and heterogeneity of the ILC family	4.07E-03	6.25
Platelet-mediated interactions with vascular and circulating cells	1.15E-03	11.76
LTF danger signal response pathway	2.71E-05	15.00
Cancer immunotherapy by PD-1 blockade	2.30E-03	8.33
Pathogenesis of SARS-CoV-2 Mediated by nsp9/nsp10 Complex	1.93E-03	9.09
COVID-19 AOP	9.07E-08	26.67
Cytokines and Inflammatory Response	2.91E-03	7.41
Type II interferon signaling (IFNG)	5.42E-03	5.41

### Topological characteristics of the PCLMN and subcellular localization of key lncRNAs

**Figure 3:**
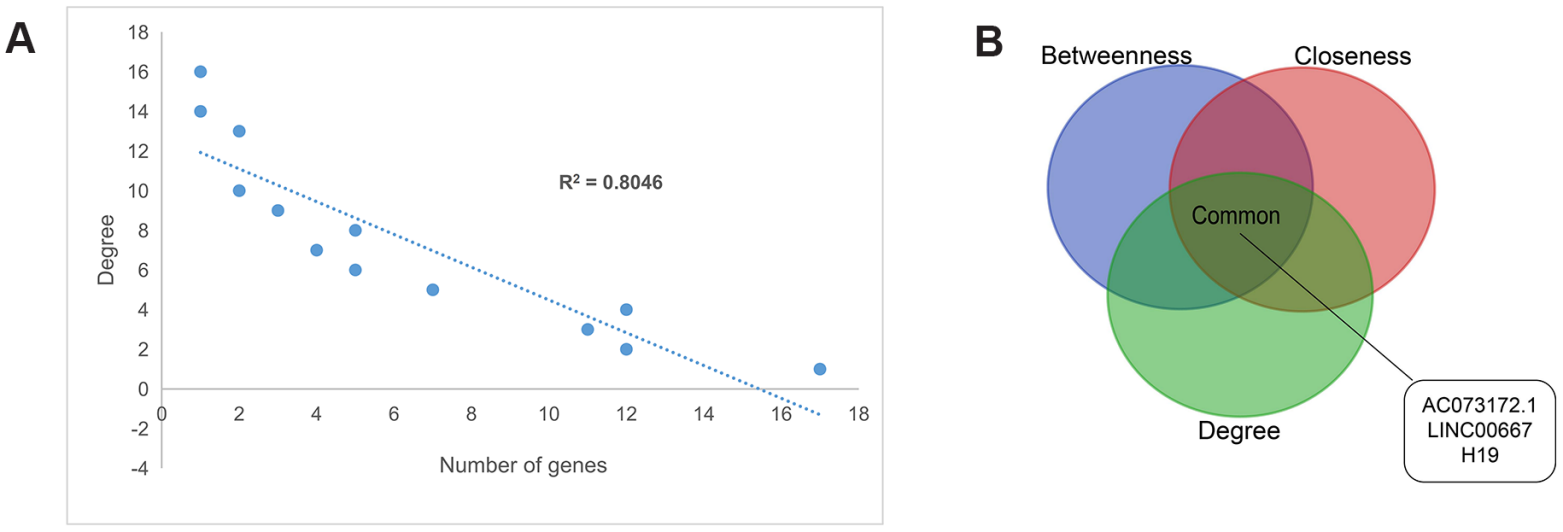
**Topology features of the PCLMN.** (**A**) Degree distributions of the network. All degrees followed a power-law distribution. (**B**) Identification of 3 lncRNA genes simultaneously ranking within the top-10 lists across degree, betweenness, and closeness.

The degree distribution of the PCLMN was examined and all the nodes were found to conform to a power-law distribution (R^2^ = 0.80), indicating that the network was scale-free (Figure 3A). Next, we analyzed the topological characteristics of the PCLMN to predict the biological functions of the network’s lncRNAs. Centralization metrics of degree, betweenness, and closeness were calculated. The top ten hits for each topological parameter were then selected, and three lncRNAs, i.e. LINC00667, AC073172.1, and H19, were found to intersect across the three features (Figure 3B).

**Figure 4:**
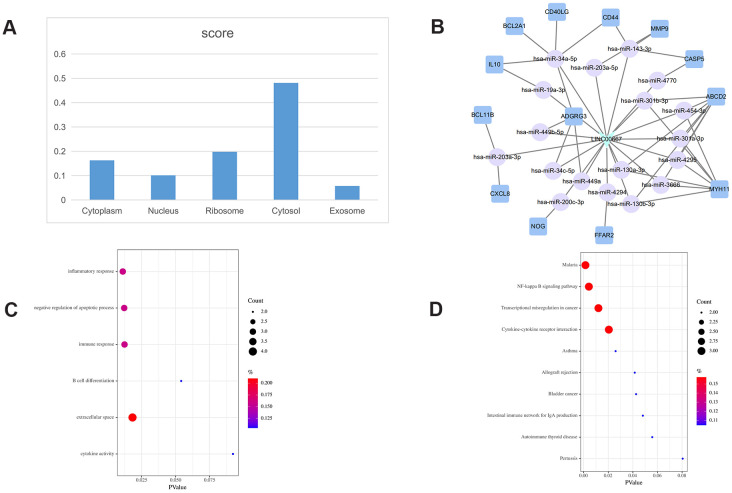
**LINC00667-related ceRNA sub-network analysis.** (**A**) Subcellular location analysis for LINC00667. (**B**) ceRNA network of LINC00667. (**C**) GO biological process enrichment results for LINC00667. (**D**) KEGG pathways enriched in LINC00667.

**Figure 5:**
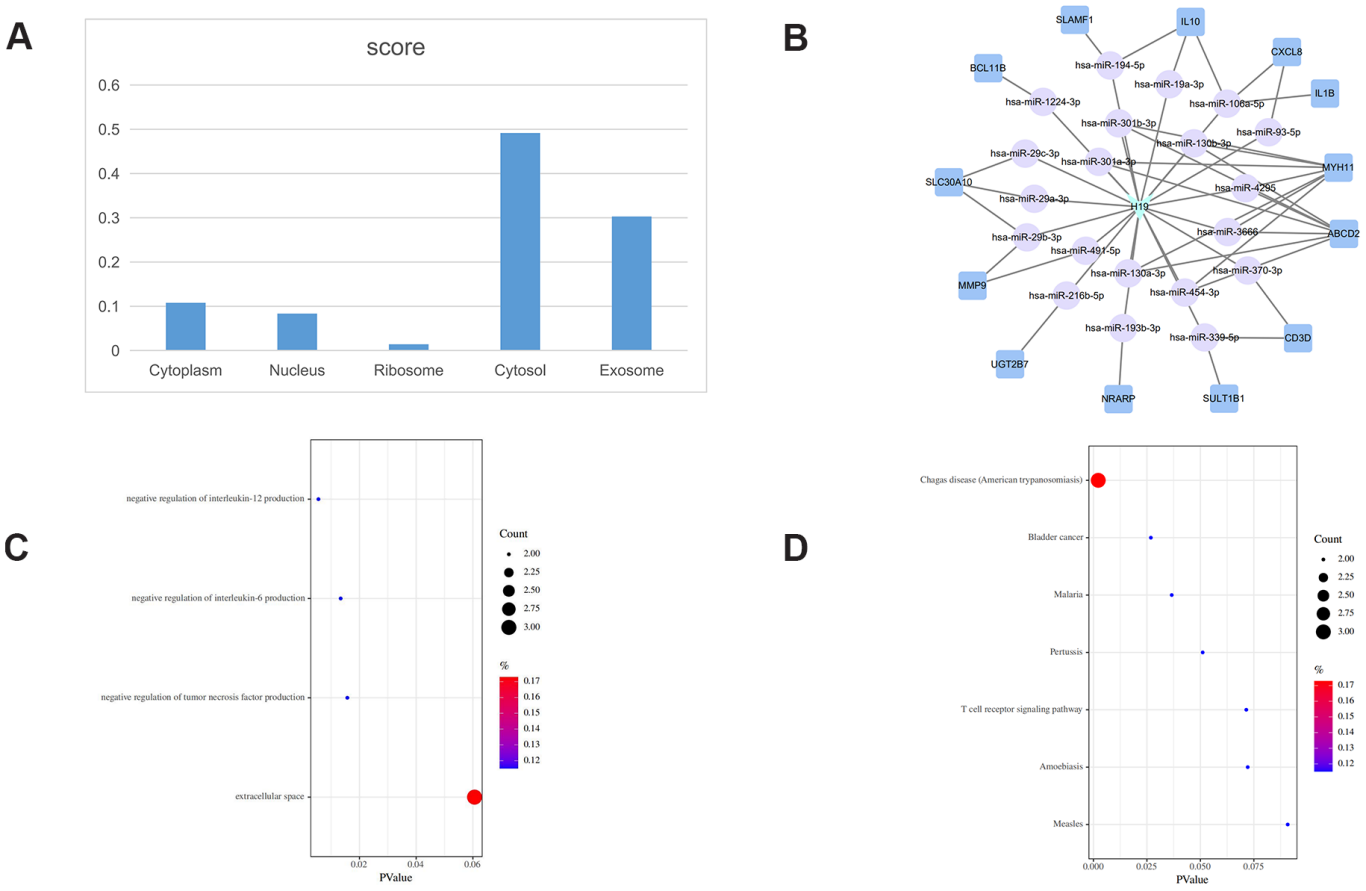
**H19-related ceRNA sub-network.** (**A**) Subcellular location analysis for H19. (**B**) ceRNA network of H19. (**C**) GO biological process enrichment for H19. (**D**) KEGG pathways enriched in H19.

**Figure 6:**
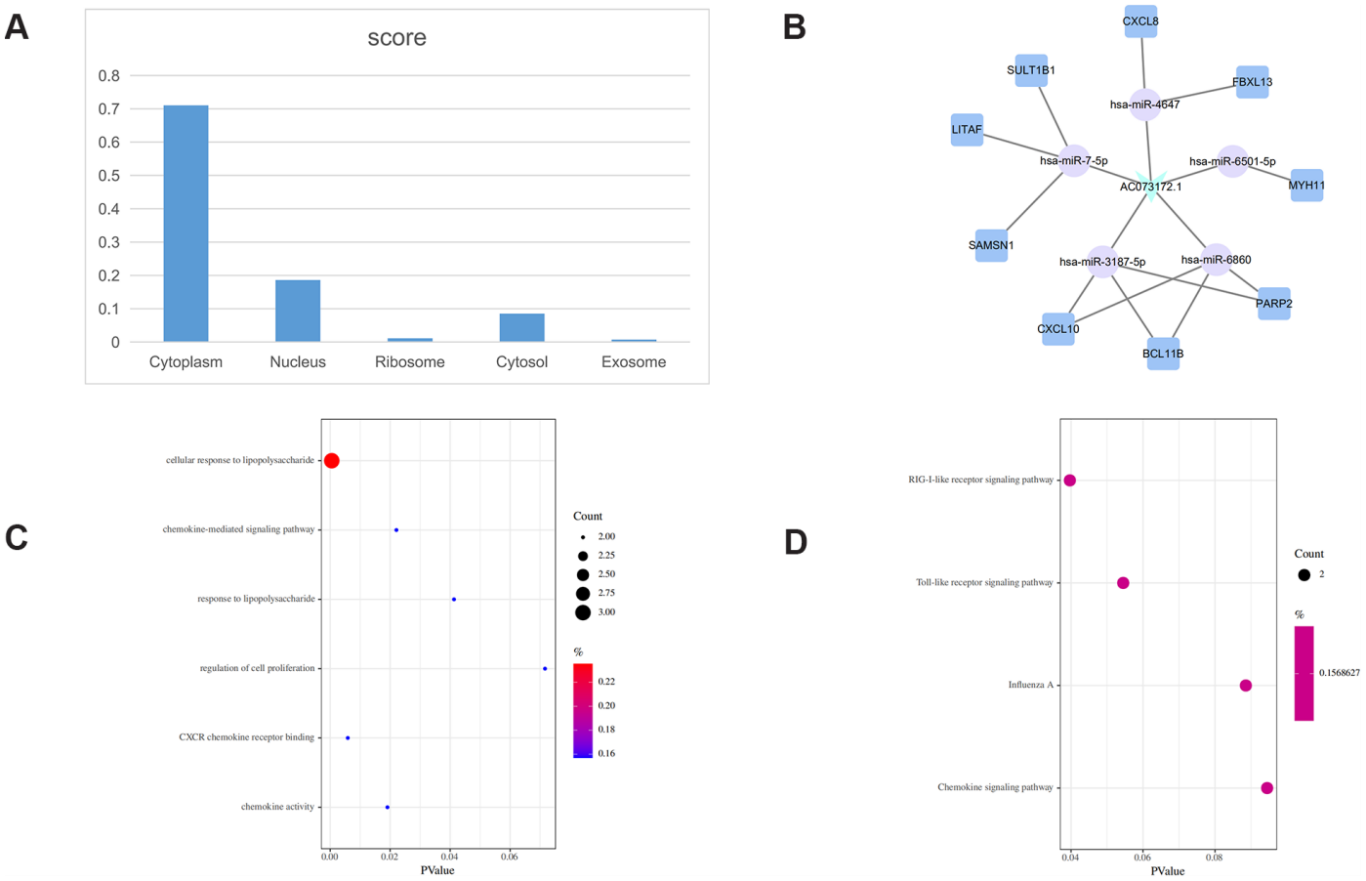
**AC073172.1-related ceRNA sub-network.** (**A**) Subcellular location analysis for AC073172.1. (**B**) ceRNA network of AC073172.1. (**C**) GO biological process enrichment analysis for AC073172.1. (**D**) KEGG pathways enriched in AC073172.1.

The subcellular localization of lncRNAs provides important clues about their potential functions. To explore the subcellular localization of the key lncRNAs identified in the PCLMN, we accessed two online platforms, i.e. LncLocator and RNAlocate, and verified the results against iLoc-lncRNA, a sequence-based subcellular localization prediction tool. Summary analysis results for LINC00667, AC073172.1, and H19 are displayed in Figures 4–6, respectively. LINC00667 showed a predominant cytosolic localization, confirmed also by iLoc-LncRNA (probability score = 0.853 for ‘cytosol/cytoplasm’) (Figure 4A). As shown in Figure 4B, there were 13 nearest mRNA neighbors for LINC00667 in the PCLMN. GO analysis indicated significant associations of LINC00667 with 4 GO terms under Biological Process (BP), one term under Molecular Function (MF), and one term under Cellular Component (CC) (Figure 4C). Meanwhile, 10 KEGG pathways showed significant enrichment in LINC00667 (Figure 4D).

H19 localized also mainly to the cytoplasm (Figure 5A), with iLoc-LncRNA yielding a probability score of 0.603 for the ‘cytosol/cytoplasm’ term. Meanwhile, 13 mRNAs were identified as nearest mRNA neighbors of H19 in the PCLMN (Figure 5B). GO classification detected enrichment by H19 in 3 terms under BP and one term under CC (Figure 5C). H19 was also enriched in 7 KEGG pathways, as shown in Figure 5D.

AC073172.1 showed also a predominant cytoplasmic localization (Figure 6A), with the iLoc-LncRNA tool indicating a probability score of 0.868 for the ‘cytosol/cytoplasm’ category. Seventeen mRNAs were detected as the nearest mRNA neighbors of H19 in the PCLMN (Figure 6B). Upon GO classification, 4 BP and 2 MF terms were found to be enriched in H19 (Figure 6C). In turn, 4 enriched KEGG pathways were identified for this lncRNA (Figure 6D).

### Identification of putative transcription factors regulating key lncRNAs in the PCLMN

**Figure 7:**
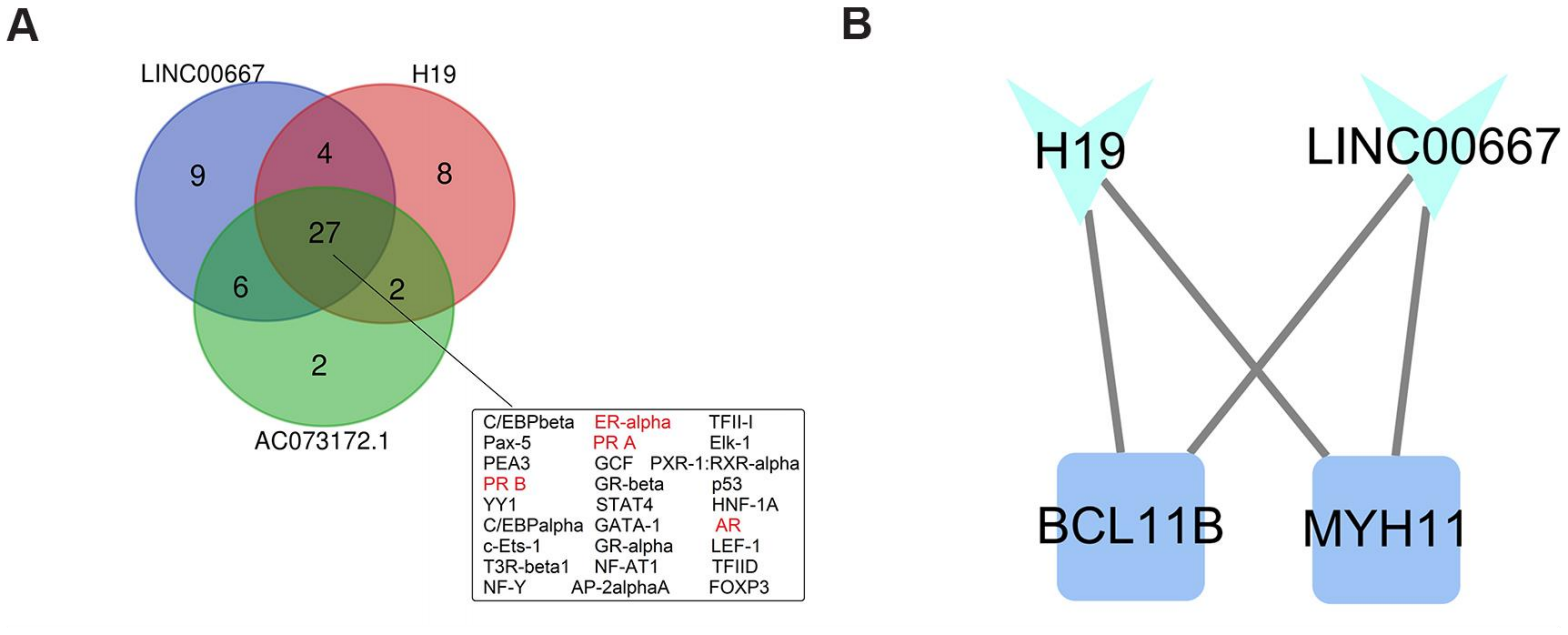
**Identification of putative transcription factors and module analysis.** (**A**) Venn diagram of predicted transcription factors (TFs) regulating the expression of the three key lncRNAs in the PCMLN. Among 27 TFs associated with all three lncRNAs, four (marked in red) are known regulators of the expression of sexual hormones. (**B**) Representation of the functional module identified in the PCLMN.

Promoter sequence-based prediction of candidate regulatory TFs of the key lncRNAs was carried out using PROMO software with maximum matrix dissimilarity rate < 3. A total of 27 common TFs targeting the promoters of the 3 key lncRNAs were identified (Figure 7A). Of these, 4 TFs, including ER-alpha, PRA, PRB, and AR (marked in red in Figure 7A), had been previously associated with PCOS.

### Identification of functional modules in the PCLMN

We performed functional module prediction on the PCLMN using the MCODE clustering algorithm implemented in Cytoscape. A functional module containing H19, LINC00667, and two protein-coding genes, namely MYH11 (which encodes the smooth muscle myosin heavy chain) and BCL11B (which encodes a zinc finger protein TF) was thus identified (Figure 7B).

## DISCUSSION

PCOS represents a prevalent disorder of follicular development characterized by excessive early follicular growth, arrested growth of antral follicles, and disrupted dominant follicle selection. In this study we performed a network-based computational analysis to identify and characterize lncRNAs potentially influencing PCOS. RNA/RNA interaction data from starBase, lncRNASNP2, and miRTarBase databases were used to construct a global lncRNA-miRNA-mRNA network based on the ceRNA theory. Then, a PCOS-related lncRNA-mRNA network (PCLMN) containing 41 mRNA nodes, 41 lncRNA nodes, and 203 edges was extracted by mapping DEGs in the GSE95728 dataset into the global triple network. The resulting PCLMN was then analyzed to identify putative functional biomarkers for PCOS.

GO classification and KEGG pathway analysis showed that many inflammation-related sub-categories, including ‘cytokines and inflammatory response’, ‘chemokine production’, ‘neutrophil chemotaxis’, ‘leukocyte aggregation’, ‘interleukin-10 signaling pathway’, and ‘IL-17 signaling pathway’ were enriched in the PCLMN. Previous studies underscored the contribution of chronic, low-grade inflammation to the occurrence of IR, hyperandrogenemia, and metabolic syndrome in PCOS [17, 18]. IR is highly prevalent in women with POCS and may be induced by pro-inflammatory cytokines acting directly on insulin-like receptor molecules [19]. A study found that increased serum CRP and IL-6 levels correlated with elevated insulin levels and higher insulin resistance index (HOMA-IR) scores, further suggesting that inflammatory factors are closely related to IR [20]. Along these lines, it was reported that inflammatory factor-mediated signaling inhibits the tyrosine kinase activity of the insulin receptor and interferes with insulin substrate 1 (IRS-1) synthesis, thus leading to IR by blocking insulin-dependent signaling [21]. In this regard, it was reported that α-trinositol (D-myo-inositol-1,2,6-trisphosphate), a synthetic inositol phosphate analog with significant anti-inflammatory properties [22] showed good safety and efficacy in decreasing IR in PCOS patients by acting as an insulin-sensitizer [23–26].

’Regulation of reactive oxygen species biosynthetic process’ was another GO term enriched in our PCLMN. Common features of oxidative stress in PCOS patients include increased lipid peroxidation and protein hydroxyl content and decreased antioxidant capacity [27]. These changes, paralleled and induced by exacerbated cellular production of reactive oxygen species, contribute to a proinflammatory state conducive to IR and hyperandrogenism [28].

Several immune-related biological processes and pathways, including ‘T cell selection’, ‘T cell differentiation in thymus’, ‘regulation of cytokine production involved in immune response’, ‘regulation of lymphocyte apoptotic process’, and ‘IL1 and megakaryocytes in obesity’ were also enriched in the PCLMN. Ovulation disorder is one of the common clinical manifestations and the main cause of infertility in women affected by PCOS. Several leukocyte subsets participate in the regulation of follicle growth and maturation. Lymphocytes have a promoting role on follicle growth, ovulation, and luteinization; however, abnormal inflammatory conditions can cause lymphocytes to oversecrete pro-inflammatory factors that trigger cytotoxicity and induce follicular apoptosis. This in turn leads to phagocytosis by macrophages, follicular development stagnation, and ovulation failure [29].

We further performed network topology analysis and run the MCODE clustering algorithm to identify crucial lncRNAs with central topology features in the PCLMN. We identified 3 lncRNAs (LINC00667, AC073172.1, and H19) with potentially crucial roles in regulating key pathways in PCOS. The NF-kappa B signaling pathway was enriched in the ceRNA sub-network of LINC00667. NF-kB is an important TF that initiates and regulates the expression of various inflammatory mediators, modulates the development of inflammatory responses, and is also closely involved in IR [30, 31]. Suggesting yet another link between PCOS and chronic inflammation, increased serum NF-κB levels have been reported in PCOS patients [32]. Indeed, the main functional module identified in our PCLMN contained, along with LINC00667 and H19, BCL11B, a TF expressed by all T-cell subsets that was shown to enhance TCR/CD28-triggered NF-kB activation [33].

Another biological process enriched in PCLMN genes was ‘negative regulation of apoptotic process’. Apoptosis is the mechanism responsible for follicular atresia and the basis for the cyclical growth and regression of human ovarian follicles [34]. Abnormal regulation of apoptosis has been suggested as a central mechanism of implantation failure by a previous ceRNA network analysis [35]. A recent study suggested also that reduced serum levels of caspase 9, an apoptotic marker, might be correlated to the pathogenesis of PCOS [36]. Moreover, compared with normally ovulating women, reduced expression of caspases 3, 8, and 9 and overexpression of the anti-apoptotic regulators cIAP-2 and Hsp27 were detected in oocytes of women with PCOS [37, 38].

The GO term ’negative regulation of tumor necrosis factor production’ was enriched in the ceRNA sub-network of H19. Tumor necrosis factor-α (TNF-α) is a crucial mediator of IR through its capacity to weaken the tyrosine kinase activity of the insulin receptor [19]. Expression levels of both TNF-α and TNF-α receptor 2 are upregulated during obesity and correlate with hyperinsulinemia [39]. Indeed, serum levels of TNF-α were found to be significantly elevated in PCOS patients with BMI > 27, compared to matched healthy controls [40].

The BP GO terms ‘cellular response to lipopolysaccharide’, and ‘Toll-like receptor signaling pathway’ were enriched in the ceRNA sub-network of AC073172.1. Lipopolysaccharide (LPS) is the main pathogenic component of gram-negative bacteria and a powerful inducer of inflammatory responses. LPS binds to Toll-like receptor 4 (TLR4) to activate NF-kB, promoting the transcription of TNF-α, IL-1β, and IL-6 [41]. Notably, saturated fatty acids can also activate TLR2 and TLR4 signaling and lead to IR [42]. In this regard, a recent study found that lipid-induced LPS-mediated inflammation through TLR4 is associated with obesity and worsened by PCOS [43]. On the other hand, recent studies suggested that single-nucleotide polymorphisms identified in TLR2 and TLR4 genes in PCOS patients might influence metabolic variables and increase susceptibility to PCOS [44, 45].

Finally, we identified several TFs that might regulate the expression of the 3 key lncRNAs identified in our PCMLN. Four TFs (ER-alpha, PRA, PRB, and AR) involved in sex steroid functions were predicted to have high binding affinity to transcriptional control elements in the DNA sequences of the 3 lncRNAs. Sex steroid hormones play a fundamental role in fertility by regulating reproductive function in the ovary through specific nuclear receptors. Several studies indicated that lncRNAs can synergize with TFs to regulate sex steroid functions. For example, a regulatory circuit composed of androgen receptors and PlncRNA-1 was shown to promote prostate cancer [46]. Another lncRNA, HOTAIR, is a direct target of ER-mediated transcriptional repression and its upregulation promotes ligand-independent ER activities [47]. Estrogens act by binding to estrogen receptor-alpha (ERα) and beta (ERβ) [48], both of which are expressed in the human ovary [49]. Studies in mice showed that ERα knockout leads to a PCOS phenotype defined by the presence of polycystic ovaries and increased luteinizing hormone (LH) levels, present impaired glucose tolerance, and eventually develop IR [50, 51]. In these mice, such condition was aggravated by accumulation of bioactive lipid intermediates and inflammation became more severe upon high-fat feeding, suggesting that ERα is also essential to protect against tissue inflammation [52]. The progesterone receptor (PR) and the androgen receptor (AR) belong to the nuclear hormone receptor family, which are associated with the regulation of eukaryotic gene expression and influence cellular proliferation and differentiation in target tissues. A previous developmental study on PRA- and PRB-knockout mice suggested that PRA is necessary for ovulation [53]. PRA and PRB are expressed in human granulosa cells and were found to be significantly downregulated in PCOS patients [54]. Human *in vitro* and *in vivo* studies found that the number of CAG repeats, which encode for an amino-acid sequence in the receptor’s transactivation domain, associates inversely the AR activity [55]. It was reported that short CAG repeats were more frequent in PCOS, possibly eliciting androgenic effects, while longer CAG repeats were more recurrent in the control group, involving probably a protective effect [56].

In summary, our study unmasked a regulatory network involving novel interactions between lncRNAs, sex steroids, and TFs with potential influence in the occurrence and development of PCOS. Further functional studies are warranted to validate the present findings and to explore their therapeutic implications.

## MATERIALS AND METHODS

### miRNA-lncRNA and miRNA-mRNA interaction data

Predicted lncRNA-miRNA pairs were obtained from starBase V3.0 [57] and lncRNASNP2 [58] databases. The latter was also used for predicting potential miRNAs targeted by DELs. Predicted miRNA-mRNA pairs were in turn obtained from miRTarBase [59] and starbase V3.0. Next, the global triple network was constructed as the background network to identify gene interactions.

### Gene expression profile

Gene expression data from the GSE95728 dataset, based on the GPL16956 platform (Agilent-045997 Arraystar human lncRNA microarray V3), was downloaded from the Gene Expression Omnibus (GEO) database. GSE95728 included lncRNA and mRNA expression profiles from granulosa cells from seven PCOS patients and seven controls (women with normal ovarian reserve).

### Differential gene expression analysis and probe re-annotation

Expression data was imported into R-studio (https://rstudio.com) and normalized with the RMA algorithm [60]. Bioconductor’s limma package was applied to identify DEGs between control and PCOS samples, based on |log2(fold change)| > 2 and adjusted P <0.05. Probe annotation data provided by Agilent were aligned to both human long non-coding and protein-coding transcript sequences retrieved from the GENCODE database by running the SeqMap program [61]. Alignment results were filtered as follows: 1) probes matched to one transcript were retained, whereas probes simultaneously matched to protein-coding and long non-coding transcripts were deleted. Two sets of probes-transcripts pairs were finally obtained; 2) for each probe–transcript pair, probes matched to more than one transcript were removed; 3) transcripts were finally selected if they matched at least three probes.

### Construction of the PCOS-related lncRNA-mRNA network

To construct the PCLMN, we mapped all the DELs and DEMs into the global triple network. Then, lncRNA-miRNA-mRNA interactions were extracted by hypergeometric test with P < 0.01. The p-value was measured as:

$$P=1-\sum_{i=0}^{r-1}\frac{\left(\frac{t}{i}\right)\left(\frac{m\;-\;t}{n\;-\;i}\right)}{\left(\frac{m}{n}\right)}$$

where *m* represents the total number of miRNAs in miRTarBase and starbase V3.0, *t* denotes the number of miRNAs interacting with an mRNA, *n* indicates the number of miRNAs interacting with a lncRNA, and *r* represents the number of miRNAs shared between the mRNA/lncRNA pair.

### Hierarchical clustering

Genes with comparable expression profiles were grouped by unsupervised hierarchical clustering using Multiple Experiment Viewer (MeV V4.9) software. The data were normalized and processed using Pearson’s correlation as distance metric and average linkage clustering algorithm.

### Enrichment analysis

Functional analysis of DEGs in the PCLMN and in the ceRNA sub-networks was performed using Gene Ontology (GO) and Kyoto Encyclopedia of Genes and Genomes (KEGG) pathway analyses using DAVID V6.8 and Cytoscape V3.8.0 with the ClueGo V2.3.7 plug-in. P values were calculated by two-sided hypergeometric test and Benjamini-Hochberg adjustment. GO terms and KEGG pathways with p <0.05 were considered statistically significant. Results were visualized with Cytoscape.

### Topological analysis and selection of key lncRNAs

We performed topological analysis of DELs DEMs to identify the central nodes of the PCLMN network. Topological parameters, including closeness, betweenness, and degree, were assessed using Cytoscape with the CentiScaPe V2.2 plug-in. The top 10 genes ranked by each measure were compared and those overlapping across all three topological parameters were chosen as key genes for further ceRNA analysis.

### Subcellular localization analysis

We predicted the subcellular localization of key lncRNAs via LncLocator [62], a public platform based on a stacked ensemble classifier. The lncRNA subcellular localization information used in LncLocator, backed by experimental evidence, was extracted from RNAlocate database (http://www.rnasociety.org/rnalocate). Furthermore, we used iLoc-lncRNA, a sequence-based tool for subcellular localization prediction, to validate the above results [63]. The sequences of the key lncRNAs were downloaded from UCSC Genome Browser database.

### Construction of ceRNA sub-networks

We extracted all the key lncRNAs and their nearest mRNA neighbors from transcript clusters in the PCLMN. miRNAs associated with lncRNA/mRNA pairs were also extracted from the global triple network and used to identify candidate lncRNA-miRNA-mRNA triplets. Then we constructed the ceRNA networks based on the ceRNA theory and visualized them with Cytoscape software.

### Identification of putative transcription factors

Transcription factors (TFs) can bind to the DNA regulatory elements of lncRNAs to activate or inhibit their expression. To assess potential linkages between the key lncRNAs, we identified the TFs that might regulate them. Promoters were defined as DNA regions within 2 kb upstream of lncRNA transcriptional start sites. We used PROMO V3.0.2 software with maximum matrix dissimilarity rate < 3 to scan the predicted TFs [64, 65], and then used a Venn diagram to identify overlapping TFs targeting all the key lncRNAs.

### Identification of functional modules in the PCLMN

LncRNAs participate in biological processes as members of functional modules encompassing other genes. To explore lncRNA-related functional modules in our network, we run Cytoscape with the MCODE plug-in applying the “Haircut,” “Fluff,” and Node Score Cutoff: 0.2 options.
